# In Vivo Glycan Engineering via the Mannosidase I Inhibitor (Kifunensine) Improves Efficacy of Rituximab Manufactured in *Nicotiana benthamiana* Plants

**DOI:** 10.3390/ijms20010194

**Published:** 2019-01-07

**Authors:** Vally Kommineni, Matthew Markert, Zhongjie Ren, Sreenath Palle, Berenice Carrillo, Jasmine Deng, Armando Tejeda, Somen Nandi, Karen A. McDonald, Sylvain Marcel, Barry Holtz

**Affiliations:** 1 iBio CDMO, LLC, 8800 Health Science Center Parkway, Bryan, TX 77807, USA; mmarkert@ibiocmo.com (M.M.); zren@ibiocmo.com (Z.R.); spalle@ibiocmo.com (S.P.); bcarrillo@ibiocmo.com (B.C.); jdeng@ibiocmo.com (J.D.); atejeda@ibiocmo.com (A.T.); smarcel@ibiocmo.com (S.M.); bholtz@ibioinc.com (B.H.); 2 Global HealthShare® Initiative, University of California at Davis, Davis, CA 95616, USA; snandi@ucdavis.edu (S.N.); kamcdonald@ucdavis.edu (K.A.M.); 3 Department of Chemical Engineering, University of California at Davis, Davis, CA 95616, USA

**Keywords:** ADCC, glycosylation, kifunensine, plant made pharmaceuticals, monoclonal antibody

## Abstract

N-glycosylation has been shown to affect the pharmacokinetic properties of several classes of biologics, including monoclonal antibodies, blood factors, and lysosomal enzymes. In the last two decades, N-glycan engineering has been employed to achieve a N-glycosylation profile that is either more consistent or aligned with a specific improved activity (i.e., effector function or serum half-life). In particular, attention has focused on engineering processes in vivo or in vitro to alter the structure of the N-glycosylation of the Fc region of anti-cancer monoclonal antibodies in order to increase antibody-dependent cell-mediated cytotoxicity (ADCC). Here, we applied the mannosidase I inhibitor kifunensine to the *Nicotiana benthamiana* transient expression platform to produce an afucosylated anti-CD20 antibody (rituximab). We determined the optimal concentration of kifunensine used in the infiltration solution, 0.375 µM, which was sufficient to produce exclusively oligomannose glycoforms, at a concentration 14 times lower than previously published levels. The resulting afucosylated rituximab revealed a 14-fold increase in ADCC activity targeting the lymphoma cell line Wil2-S when compared with rituximab produced in the absence of kifunensine. When applied to the cost-effective and scalable *N. benthamiana* transient expression platform, the use of kifunensine allows simple in-process glycan engineering without the need for transgenic hosts.

## 1. Introduction

Monoclonal antibodies (mAbs) represent the fastest growing class of therapeutics [1,2] and have been especially beneficial in the treatment of cancer [3]. Since the approval of the first anti-cancer monoclonal antibody in 1986 [4], several innovations have improved the potency of monoclonal antibodies used in immunotherapies that offer increased drug efficiency and/or lower drug dosage for a specific treatment. Among them, glycan engineering of the oligosaccharides attached to Asn297 of the Fc region of the heavy chain has been shown to affect antibody-dependent cell-mediated cytotoxicity (ADCC), complement dependent cytotoxicity (CDC), and binding to the neonatal Fc receptor, FcRn [5,6,7]. Specific oligosaccharides influence the affinity of the antibody Fc domain to Fc receptor present on effector cells resulting in altered biological functions. For example, the removal of terminal galactose residues on mammalian cell-derived antibodies lowered C1q binding [8,9], while ADCC activity is almost completely dependent on the presence or absence of fucose residues bound to the glycosylation core [10,11,12]. Several approaches have been employed to manufacture a monoclonal antibody with a decreased or absent core fucosylation. One strategy is to use cell lines or organisms with modified glycosylation pathways [13,14,15,16]. The alteration of the expression of key enzymes in the host glycosylation pathway such as the mammalian α1,6-fucosyltransferase [15], the plant α1,3-fucosyltransferase [17,18,19,20,21], the GDP (guanosine 5’-diphosphate)-mannose 4,6-dehydratase [11,22], or the β1,4-N-Acetylglucosaminyltransferase III [14,23] led to afucosylated antibodies with improved anti-tumor activity. This led to the approval of mogamulizumab (POTELIGIO^®^, Kyowa Hakko Kirin) [24] and obinutuzumab (Gazyva^®^, Roche) [25] in 2012 and 2013, respectively, both produced in glycoengineered mammalian cell lines. Another approach to alter the antibody glycosylation profile is to modify the culture conditions of the host cells by adjusting the growth environment [26] or supplementing the media with inhibitors of enzymes in the glycosylation pathway such as N-butyldeoxynojirimycin (NB-DNJ), mannostatin A, swainsonine, or kifunensine [27,28,29,30,31]. Kifunensine from the actinomycete *Kitasatosporia kifunense* 9482 inhibits class I α-mannosidases and blocks N-glycan synthesis at the Man8GlcNAc2 (Man_8_) or Man9GlcNAc2 (Man_9_) stage before the core fucose is added [28,32]. In mammalian cell culture, kifunensine was successfully employed to produce protein with >90% high-mannose content [28,29,33,34]. This effect was similar across many different proteins including antibodies, suggesting that this simple treatment could be applied broadly. Compared to other α-mannosidase I inhibitors, kifunensine is highly effective on mammalian cell culture without significantly affecting cell growth or protein yield, even at concentrations as low as 100 ng/mL culture [29,33,34]. Similar to mammalian cell studies, kifunensine was used in conjunction with the *Nicotiana benthamiana* transient protein expression systems to produce proteins with >98% afucosylated high-mannose glycans [35,36,37]. In plants, the non-human α1,3-fucose and β1,2-xylose residues are commonly added in the Golgi apparatus after mannose trimming by mannosidases in the endoplasmic reticulum. Upon kifunensine treatment, addition of α1,3-fucose and β1,2-xylose residues were not observed on the Man_3_ to Man_9_ structures [35,37]. However, the amount of kifunensine used in these studies was at or above 1.16 µg/mL (5 µM) [35,37], which significantly increases production costs at the manufacturing scale.

Kifunensine is currently being used to manufacture a recombinant glucocerebrosidase in HT1080 fibrosarcoma cells (Velaglucerase alpha, Shire Plc.) to treat type 1 Gaucher disease [38]. The process of Velaglucerase alpha was design to modify the glycosylation profile of the protein toward oligomannose N-glycans to improve mannose-receptor mediated uptake of the drug into macrophages, the target cells [39]. Here, we determined the optimal concentration of kifunensine and demonstrate that kifunensine addition at a concentration of 0.375 µM (87 ng/mL) in the *Agrobacterium* infiltration solution of *N. benthamiana* plants during the vacuum infiltration process allows the production of exclusively high-mannose recombinant proteins. The anti-CD20 monoclonal antibody rituximab, approved for the treatment of non-Hodgkin’s lymphoma (NHL) [40], was selected to evaluate the effectiveness of kifunensine for the production of an anti-cancer antibody with enhanced antibody-dependent cell mediated cytotoxicity (ADCC). ADCC efficacy of rituximab is inversely correlated with the content of core fucose [41,42], suggesting that a rituximab variant with altered glycosylation would lower dosing requirements. More importantly, we demonstrate that afucosylated high-mannose decorated antibody, derived from the treated plants, exhibits increased ADCC effector function, as compared with rituximab derived from non-treated plants. The increased ADCC activity was verified using effector cells carrying both FcγRIIIa-V158 and FcγRIIIa-F158 allotypes. Several strategies could be implemented to modulate the plant-specific glycans: (i) Protein containment in the Endoplasmic Reticulum (ER) using specific signal sequences (i.e., SEKDEL sequence: Ser-Glu-Lys-Asp-Glu-Leu) [43], (ii) knockdown of fucosyltransferase and xylosyltransferase enzymes in *N. benthamiana* with RNA interference (RNAi) technology [18], (iii) knockout of fucosyltransferase and xylosyltransferase enzymes in *N. benthamiana* using gene editing [44], and (iv) replacement of plant glycans with human glycans through glyco-remodeling [45]. Gene editing using sequence-specific transcription-activator-like effector nucleases (TALENs) was only partially effective [44], while the use of transgenic knockdown lines at manufacturing require more exigent containment and cleaning procedures. Our approach eliminates the need for modification of the primary sequence or the use of transgenic, regulated material for manufacturing. Combined with the scalability [46] and low manufacturing cost associated with the *N. benthamiana* transient expression system [47], this method represents an excellent alternative to the use of either glycoengineered or kifunensine-treated mammalian cell lines for the production of afucosylated anti-cancer antibody. 

## 2. Results

### 2.1. Kifunensine Treatment of N. benthamiana did not Impede Rituximab Expression

**Figure 1:**
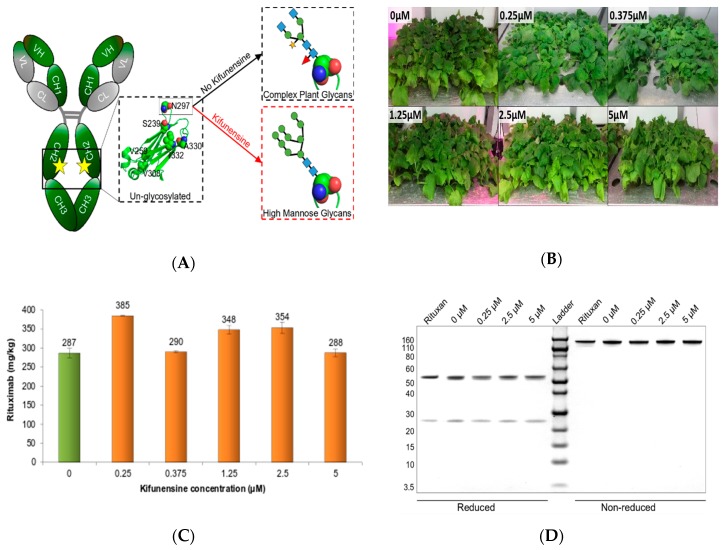
Effect of kifunensine on plant-made rituximab. (**A**) Schematic representation of Immunoglobulin G 1 (IgG1) glycosylation. Complex-type plant glycans (black dotted lines) formed in the absence of kifunensine (black arrow) transformed into Oligomannose-type glycans (red dotted lines) in the presence of kifunensine (red arrow). The oligosaccharide structures are shown in the symbolic depiction suggested by the Consortium of Functional Glycomics (www.functionalglycomics.org). Blue squares -N-acetylglucosamine; Green circles -Mannose; Orange Star- Xylose; and Red Triangle-Fucose. (**B**) Phenotype of *N. benthamiana* plants infiltrated under vacuum with *Agrobacterium* suspension ± kifunensine. Each experimental group received different concentrations of kifunensine in the *Agrobacterium* infiltration solution and concentrations are indicated on top of each treatment image. (**C**) Quantification of rituximab in crude protein extracts using Biolayer interferometry (BLItz^®^, ForteBio). Expression levels of rituximab in 7 dpi plant extracts with (orange) and without (green) kifinensine are reported in mg rituximab/kg fresh weight (FW). Error bars represent standard deviations of duplicated expression measurements, where *n* = 3. (**D**) SDS-PAGE (sodium dodecyl sulfate Polyacrylamide gel electrophoresis) analysis of purified rituximab samples under reduced and non-reduced conditions. Rituxan, plant-made rituximab with no kifunensine, 0.25 µM kifunensine, 2.5 µM kifunensine, and 5 µM kifunensine were separated on a 4–12% Bis-Tris gel along with Novex sharp pre-stained protein standard.

Rituximab transiently expressed in *N. benthamiana* typically presents complex glycans of GlcNAc_2_(Xyl)Man_3_(Fuc)GlcNAc_2_ on Asn297 of the heavy chain (Figure 1A). 

We have hypothesized that a treatment with kifunensine would inhibit trimming of mannose residues in the endoplasmic reticulum (ER), subsequently preventing the addition of α1,3-fucose and β1,2-xylose residues on the polysaccharide core (Figure 1A). To do so, 60 plants per conditions were vacuum infiltrated in a solution of *Agrobacteria* with or without kifunensine, ranging from 0 to 5 μM (0 µg/mL to 1.16 µg/mL). Visual observation of infiltrated plants from three to seven days post infiltration (dpi) revealed no noticeable phenotypic or morphological differences between treated and untreated control plants (Figure 1B). All leave and stems were collected from each infiltration (~250 g plant biomass) and pooled for protein extraction and purification. Rituximab expression levels were quantified at 7 dpi and revealed a low to moderate increase (up to 34%) in antibody expression between untreated and treated plants, with the average rituximab expression level ranging from 288 mg/kg to 385 mg/kg whole plant fresh weight (FW) in treated plants, compared to 287 mg/kg whole plant FW in untreated plants (Figure 1C). These observations demonstrated that the kifunensine treatments were not detrimental to plant growth or protein expression. To evaluate the effect of kifunensine treatments on the integrity and assembly of rituximab, SDS-PAGE analysis was carried out under reduced and non-reduced conditions. As illustrated in Figure 1D, rituximab derived from kifunensine treated and untreated conditions appeared intact and fully assembled. Non-reduced rituximab migrated at the expected molecular weight (MW) of ~145 kDa, while the reduced heavy and light chains migrated at the expected MW of ~50 kDa and ~25 kDa, respectively (Figure 1D). Infiltration experiments were run in duplicates.

### 2.2. A Concentration of 0.375 μM of Kifunensine is Sufficient to Produce Rituximab with Afucosylated, Oligomannose-Type Glycoforms Exclusively

**Figure 2:**
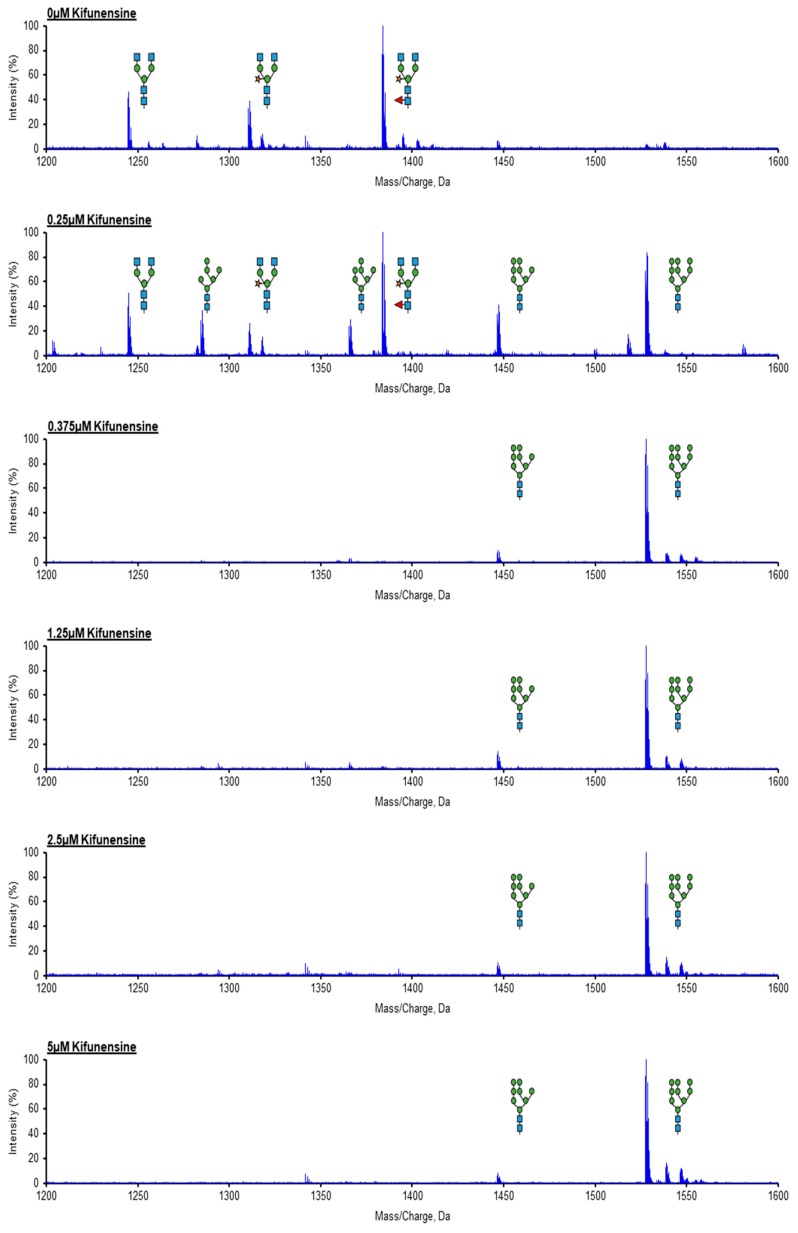
N-Glycan analysis of rituximab expressed in *N. benthamiana* plants with/without kifunensine. LC-MS (Liquid Chromatography-Mass Spectrometry) glycopeptide profiling of rituximab expressed in control and kifunensine treated plants. The distribution of glycoforms in each sample is illustrated and kifunensine concentrations are indicated on each image. Blue squares -N-acetylglucosamine; Green circles -Mannose; Orange Star- Xylose; and Red Triangle-Fucose.

The N-glycosylation profiles of purified rituximab expressed in *N. benthamiana* were evaluated by LC-MS/MS (Liquid Chromatography-Mass Spectrometry) analysis (Figure 2).

**Figure 3:**
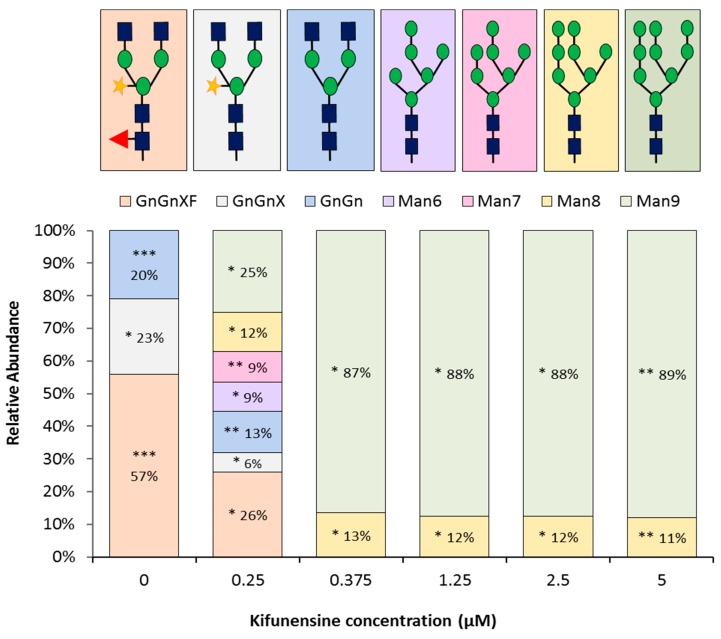
LC-MS glycopeptide profiling of rituximab samples. The ratio between oligomannose glycoforms (Man_8_, Man_9_) and hybrid glycoforms (GnGn, GnGnXF, and GnGnX) are represented in histograms. Kifunensine concentrations are indicated on the X axis and glycoform percentages are indicated on each sample. Blue squares -N-acetylglucosamine; Green circles -Mannose; Orange Star- Xylose; and Red Triangle-Fucose. Statistical analysis derived from two biological and two technical replicates. Standard deviations (SD) are indicated next to the glycan percentage as follows: * SD value 0 to 1%, ** SD value from 1 to 3%, and *** SD value from 3 to 4%.

The major glycoforms were compared based on the relative intensity of the Asn297 glycopeptide masses identified by LC-MS/MS. We first established the glycoform distribution of rituximab derived from untreated plants (Figure 2; 0 μM kifunensine). As previously described in the literature, this plant-derived rituximab control exhibited primarily complex-type N-glycans [48], with the most abundant N-glycan structure being GlcNAc_2_(Xyl)Man_3_(Fuc)GlcNAc_2_ (Figure 3).

On the other hand, there were significant differences in rituximab N-glycan profiles between untreated and treated samples. Complete conversion of plant complex glycans to oligomannose-type glycans was observed when *N. benthamiana* plants were infiltrated with ‘higher range’ and ‘medium range’ concentrations of kifunensine (from 5 μM to 0.375 μM). The GlcNAc_2_Man9 (Man_9_) and GlcNAc_2_Man8 (Man_8_) were the major glycoforms observed (Figure 2; 0.25 to 5 μM kifunensine) with Man_9_ being the most abundant. In fact, the same oligomannose-type glycoform distribution was observed whether 0.375 μM or higher kifunensine concentration was used, indicating that 0.375 μM is sufficient to provide homogeneous rituximab with oligomannose-type glycans (Figure 3). When lower concentrations of kifunensine were used, a mixture of oligomannose, hybrid, and complex glycans was detected (Figure 2; 0.25 μM kifunensine). For instance, the glycosylation profile of rituximab from plants treated with 0.25 µM contained more than ~48% hybrid/complex glycan modifications (GnGn, GnGnX, and GnGnXF) and ~52% oligomannose (Man_6_, Man_7_, Man_8_, and Man_9_) glycosylation (Figure 3). Importantly, no α1,3-fucose or β1,2-xylose residues were detected in rituximab derived from plants treated with ≥0.375 μM kifunensine.

### 2.3. Rituximab Decorated with Oligomannose Residues Binds Target CD20 as Efficiently as Rituxan^®^

**Figure 4:**
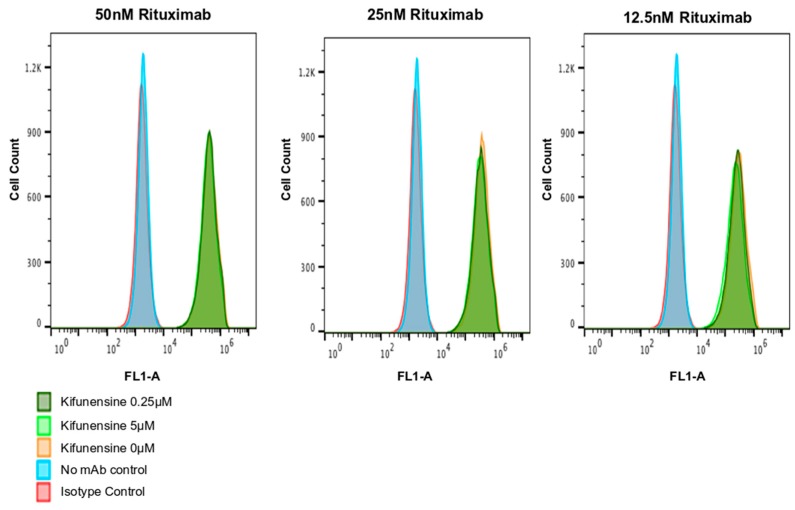
CD20 binding assay of rituximab treated with kifunensine 5 µM, 0.25 µM and untreated controls with Flow Cytometry analysis. Plant-made rituximab was used at concentrations of 50 nM, 25 nM and 12.5 nM. Antibodies bound to CD20 on Wil2-S were detected with goat anti-human IgG polyclonal antibodies conjugated with Fluorescein isothiocyanate (FITC). Median Fluorescence intensity (MFI) was derived from the median value of the fluorescence histogram.

The mode of action of rituximab is characterized first by binding to the tumor cell surface antigen CD20 present of lymphoma cells before cell cytotoxicity is induced. Thus, to confirm that rituximab derived from kifunensine-treated plants binds CD20, a flow cytometry analysis using the human B cell line Wil2-S was performed. Rituximab samples purified from plants treated with 0, 0.25, 0.375, 1.25, 2.5, and 5 µM of kifunensine was compared to Rituxan^®^ for the proteins’ ability to bind CD20 on Wil2-S cells. This analysis revealed no difference in the binding affinity of rituximab derived from plants treated with 5 μM or 0.25 μM kifunensine as compared with Rituxan^®^ (Figure 4). In all antibody independent assays and isotype controls, no CD20 binding signal was observed.

### 2.4. Effect of Kifunensine on Biological Activity of Rituximab Produced in N. benthamiana

**Figure 5:**
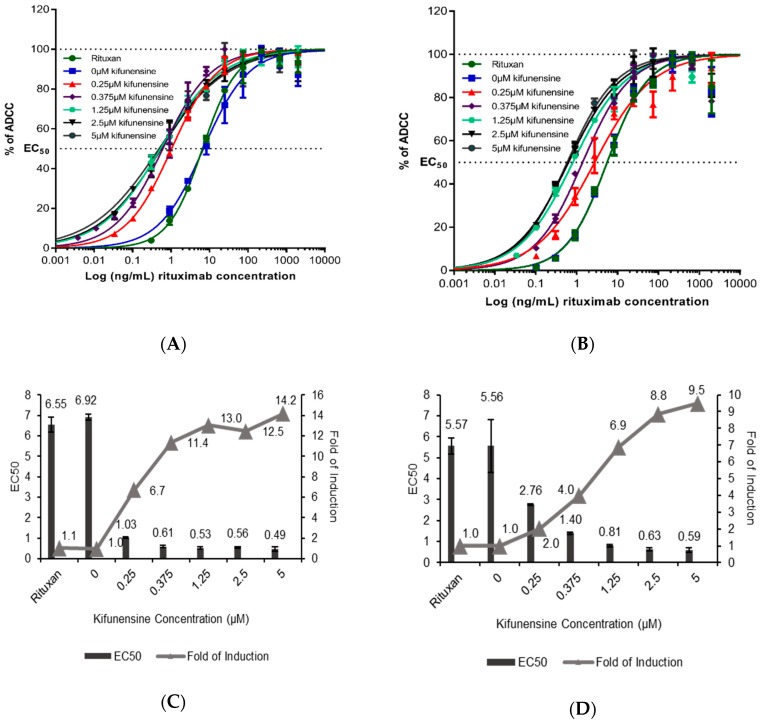
Antibody-dependent cell-mediated cytotoxicity of rituximab samples expressed in the presence or absence of kifunensine. Assay was performed using Wil2-S target cells along with either high affinity V/V 158 FcγRIIIa variant (**A**,**C**) or low affinity F/F 158 FcγRIIIa variant (**B**,**D**) engineered Jurkat cells. The effector cell: target cell ratio was 10:1. Values are expressed as normalized RLUs (**A**,**B**) and represent the mean ± Standard Deviation (SD). for triplicate analyses. Summary of ADCC activity represented as EC_50_ values. The horizontal dotted defines 100% and 50% value (**C**,**D**), normalized to the control 0 uM kifunensine control value, indicating relative activity. The error bars of each EC_50_ value correspond to the standard error of the mean.

Rituximab samples purified from plants treated with 0, 0.25, 0.375, 1.25, 2.5, and 5 µM of kifunensine, as well as a commercially available rituximab (Rituxan^®^) were assessed for their ability to generate in vitro ADCC activity against the Wil2-S human B cell line using modified Jurkat effector cells expressing either the F158 or V158 variant of the Fc receptor RIIIa (FcγRIIIa or CD16a). In the human population, FcγRIIIa polymorphism is observed at amino acid 158 [Valine (V)/Phenylalanine (F)] of the FcγRIIIa with the V158 allotype exhibiting a higher affinity for IgG1. The relative ADCC activity between the samples was represented by non-linear regression activity curves comparing normalized ADCC activities (as measured by induced florescence) to rituximab concentrations (Figure 5A,B).

A robust, dose-dependent reporter signal was produced when Wil2-S cells were co-incubated with either FcγRIIIa-V158 (Figure 5A) or FcγRIIIa-F158 (Figure 5B) effector cells and rituximab. The 50% effective concentrations (EC_50_) calculated from these curves were directly compared between samples to evaluate the efficacy of each antibody variant. Compared to rituximab from untreated plants and mammalian cell derived Rituxan^®^, rituximab derived from plants treated with ≥0.25 μM kifunensine induced significantly greater ADCC activity with both FcγRIIIa-V158 (Figure 5C) and FcγRIIIa-F158 (Figure 5D) effector cell variants. The ADCC activity of rituximab with oligomannose-type glycans was increased by 14-fold and 9.5-fold when using V158 and F158 variant effector cells, respectively (Figure 5C,D), as compared with Rituxan^®^. Interestingly, the increase in ADCC activity from rituximab derived from plants treated with 0.25 µM kifunensine (Figure 5) was found to be intermediate between the non-treated rituximab and rituximab derived from plants treated with ≥0.25 μM kifunensine (Figure 5C,D). This may be attributed to a similar intermediate content in α1,3-fucose on this rituximab sample compared to the other rituximab samples. In all antibody independent assays (AICC), no signal was observed in the absence of antibody.

## 3. Discussion

### 3.1. Kifunensine Treatment Is Well Tolerated During Expression of Rituximab in N. benthamiana

To date, several inhibitors of the N-glycosylation pathway have been used to modulate the glycosylation profiles of recombinant proteins. Among them, kifunensine, an inhibitor of class I mannosidases, acts early in the N-glycosylation processing by blocking the trimming of Man_9_ oligosaccharides to Man_5_. In comparison, application of swainsonine, an inhibitor of class II mannosidases, leads to the formation of a more heterogeneous population of N-glycans with fucose-containing hybrid structures as well as oligomannose structures [49,50]. Additionally, kifunensine has been shown to be very efficient at low concentrations, as compared with swainsonine, thus becoming a good candidate as a medium supplement for alternation of host N-glycosylation pathways. While it has been used in mammalian cells since 1990 [28], it was only recently applied in the plant expression systems [35,36,37].

In this study, we have investigated the use of kifunensine in a plant expression platform that is established for large-scale manufacturing for recombinant protein [46]. More specifically, we evaluated the concentration of kifunensine sufficient for the production of an afucosylated rituximab with enhanced biological activity. The vacuum infiltration of plants in a solution containing *Agrobacterium* culture supplemented with kifunensine at a concentration varying from 0.0625 µM to 5 µM did not affect the seven-day post-infiltration growth of the plants or the expression of rituximab. In fact, the expression of rituximab was slightly higher from kifunensine-treated plants. Similar tolerance to kifunensine treatment has also been described for the expression of antibodies in Chinese Hamster Ovary (CHO) cells [29,33]. However, it has been reported in one instance that kifunensine treatment of *N. benthamiana* plants via the growth medium led to a decrease in expression of a recombinant protein [35]. When applied during plant vacuum infiltration in the Agroinfiltration solution, kifunensine enters the interstitial spaces of the leaf tissue in contact with the host cells where recombinant protein expression occurs, rather than through uptake via the root system. The positive impact of infiltrated kifunensine on host cell tolerance and protein production may be due to its suppressing effects on the ER-associated degradation pathway [51] where proteins with trimmed oligomannose glycans may be degraded if the polysaccharide chain is not further processed or proteins transported to the Golgi apparatus [52,53].

### 3.2. Kifunensine Has a Strong Effect on the Plant N-glycosylation Machinery, even at Low Concentration

Kifunensine treatment during the agroinfiltration ultimately results in protein afucosylation as it stops mannose trimming in the endoplasmic reticulum, yielding Man_5_-Man_9_ N-glycan structures. When delivered in this fashion, a minimum concentration of 0.375 µM kifunensine was sufficient to generate rituximab harboring only oligomannose glycan structures lacking fucose residues. In agreement with reports using mammalian cell cultures [29,33], the minimum required kifunensine concentration to generate antibody devoid of fucose residues in *N. benthamiana* falls somewhere between 0.25 and 0.375 µM (58 and 87 ng/mL). Kifunensine has the practical advantage of being active at 2-to-4-fold-lower concentrations than other inhibitors of the glycosylation pathway (e.g., swainsonine or N-butyldeoxynojirimycin) making it more cost-effective. Moreover, as described here and in mammalian cell cultures, treatments with kifunensine leads to a highly homogeneous product, with ultimately no formation of core-fucosylated hybrid structures [29,33,34,35,37].

### 3.3. Kifunensine Represents a Cost-Effective Method to Produced Biobetter Anti-Cancer Antibodies

Proteins produced with glycan-engineering technologies not only lack potentially immunogenic plant-specific glycoforms, but also provide enhanced effector function. The in vitro bioassay described in this study demonstrated enhanced ADCC activity from rituximab containing high-mannose glycoforms. It is expected that the reason for this increased ADCC activity lies in the absence of fucose residues on the glycosylation core rather than the high content of mannose residues, as many studies have reported the effect of afucosylation on the ADCC activity of anti-cancer antibodies [54] including rituximab [10,20,55,56]. In fact, similar ADCC results were obtained with antibodies derived from CHO cell cultures treated with kifunensine [33,34]. It is important to note that ADCC activity was linearly proportional to the relative abundance of oligomannose glycoforms. This was particularly evident with ‘low-range’ concentrations of kifunensine applied, which generated a mixture of complex, hybrid, and oligomannose structures. With rituximab derived from a treatment of 0.25 µM kifunensine, generating a relatively small increase (24%) of oligomannose glycoforms, there was a significant but lower increase in ADCC activity (6.7-fold and 2-fold increase in ADCC activity using the FcγRIIIa-V158 and FcγRIIIa-F158 effector cell variants, respectively) (Figure 5). Importantly, plant-derived high-mannose rituximab glycoforms exhibited the same affinity for CD20 as Rituxan^®^, the commercial standard. Thus, the kifunensine treatment does not affect the paratope conformation of the plant-derived antibody. As the glycosylation profile of an anti-cancer antibody is correlated to its biological activity, it is therefore considered as a critical quality attribute that needs to be maintained during the manufacturing process [57]. To that end, the glycosylation profile of kifunensine-treated antibodies is homogeneous, consistent, and easy to control at scale, which represent a significant advantage for this technology. There are reports that antibodies carrying high mannose glycans have a shorter serum half-life, as compared with other glycoforms [33,58,59]. However, other pharmacokinetic studies with afucosylated high mannose antibodies indicated no impact on clearance [29,34,60]. Thus, the pharmacokinetics property of any antibody will have to be evaluated based on its biological activity, the target indication, and dosage regimen.

In conclusion, the application of kifunensine during transient agroinfiltration of the *N. benthamiana* host leverages the scalability and cost-effectiveness of the plant expression platform for the production of biobetter anti-cancer antibodies. First, the scale-up of rituximab expression (up to 150 kg of plant biomass but without kifunensine) was also demonstrated at the iBio CDMO facility using manufacturing procedures without affecting the expression or product quality. Second, using the process model described by Holtz et al. 2015 [46], and the findings from this study (a kifunensine concentration of 0.375 µM), the cost of cGMP (current Good Manufacturing Practice)-grade kifunensine to produce high-mannose, afucosylated antibodies at manufacturing scale (~100 kg/year of rituximab) was estimated to be less than $0.80/g of antibody produced. Thus, kifunensine can be incorporated into already established manufacturing protocols without affecting production cost significantly. Further studies will focus on determining how long the inhibition effect of kifunensine lasts during and after the plant infiltration process. This attempt to increase anti-cancer efficacy of recombinant antibodies through in-process glycan engineering represents a promising alternative to meet unmet medical needs.

## 4. Materials and Methods

### 4.1. Construction of Heavy Chain and Light Chain Expression Vectors

The genetic sequence of rituximab heavy and light chains was obtained from DrugBank (Accession DB00073). The rituximab heavy chain (HC) and light chain (LC) genes were fused to the Pathogenesis-related protein (PR1a) signal peptide sequence (UniProtKB/Swiss-Prot: P08299.1). Rituximab HC and LC genes were codon optimized for plant expression using the Nicotiana tabacum codon usage table (DNA2.0, Menlo Park, CA, USA) and independently cloned into proprietary plant viral-based expression vectors (iBio Inc., New York, NY, USA). Vectors were then mobilized in *Agrobacterium tumefaciens* strain GV3101 for transient expression in *Nicotiana benthamiana*.

### 4.2. Expression and Extraction

*Agrobacterium* clones harboring HC and LC expression vectors were grown individually in culture flasks containing Luria-Bertani (LB) medium supplemented with 50 mg/L kanamycin, 50 mg/L gentamycin and 25 mg/L rifampicin at 28 °C with agitation of 225 rpm. Cultures reaching an OD600 nm of ~3.0 were collected and diluted in 2 mM MES (2-(N-morpholino)ethanesulfonic acid) to a final OD600 nm of 0.05 for each culture. *N. benthamiana* four-week old plants, hydroponically grown under red/blue LED (Light-Emitting Diode) light were vacuum infiltrated with *Agrobacterium* culture, as described previously [46], along with varying concentrations of kifunensine mixed in the infiltration solution. Kifunensine concentrations used for infiltrations included a ‘higher range’ at 5 µM, 2.5 µM, and 1.5 µM; ‘medium range’ 1.25 µM, 0.75 µM, and 0.375 µM; and ‘lower range’ 0.25 µM, 0.125 µM, and 0.0625 µM. Control infiltration was carried out without kifunensine. Each infiltration was carried out with 60-plant batches and kifunensine was freshly prepared for each infiltration. Agro-infiltrated plants were then incubated under constant LED light at ~22 °C with relative humidity of ~ 50% for rituximab production. After seven days post-infiltration (dpi), plants were harvested, and proteins extracted. *Agrobacterium* infiltrated *N. benthamiana* biomass was mechanically homogenized in three volumes (Weight/Volume) of extraction buffer (50 mM sodium phosphate, 150 mM sodium chloride, 60 mM ascorbic acid, 5 mM EDTA, 1 mM PMSF (phenylmethylsulfonyl fluoride), pH 5.5) using a Waring blender at maximum speed for one minute. Primary clarification was achieved by centrifugation at 14,000×*g* for 20 min at 4 °C. To determine the concentration of the rituximab in the samples, the supernatant was harvested and a 4 µL sample was analyzed with the BLItz system using protein A sensors (Pall ForteBio, Menlo Park, CA, USA). After data acquisition, the concentration of rituximab in the extracts was calculated based on a rituximab reference standard.

### 4.3. Purification and SDS-PAGE Analysis

Rituximab purification was carried out as described earlier [45]. Antibody samples were analyzed on a 4–12% Bis-Tris gradient NuPAGE™ gel under reducing conditions according to the manufacturer’s protocol (Life Technologies, Carlsbad, CA, USA).

### 4.4. Intact Protein Analysis

Purified rituximab samples were exchanged into 50 mM ammonium bicarbonate, pH 8.0 buffer and adjusted to 1 mg/mL. A 5 µL aliquot was injected into a BEH C4 column 300 Å, 1.7 µm 2.1 mm × 100 mm (Waters Corporation, Milford, MA, USA) equilibrated with 0.1% (*v*/*v*) formic acid in water at a flow rate of 200 μL/min. Proteins were eluted using a gradient of 2–85% acetonitrile (*v*/*v*) over the course of 30 min. A blank solution using acetonitrile was utilized between each sample to avoid peak carry-over between runs. The mass spectrometer was calibrated using PPG (polypropylene glycol) positive ion calibration solution from SCIEX (AB SCIEX LLC, Framingham, MA, USA). The eluted proteins were introduced into the mass spectrometer and analyzed using the mass spectrometer in the positive ion mode with the following settings: Intact protein mode, scan range 1500–4000 m/z (mass to charge ratio), Accumulation Time 0.5 s, GS1 (Ion Source Gas 1) = 45, GS2 (Ion Source Gas 2) = 50, CUR (curtain gas) = 30, source temperature = 400 °C, DP (declustering potential)= 225V, CAD (collisionally activated dissociation)= 6, CE (collision energy) = 15V and ISVF (IonSpray Voltage Floating) = 4800. The raw data of intact protein analysis were processed using the software Unidec (University of Oxford, Oxford, UK) with subtract curved of, Gaussian smoothing of 5, bin every 1, peak FWHM (Full width at half maximum) of 2 and peak detection threshold of 0.1.

### 4.5. Glycopeptide Profiling

The purified rituximab samples were analyzed with LC/MS/MS based peptide mapping method as previously described [45]. The glycopeptides were identified manually by screening the N-Acetylglucosamine residue reporter ion (m/z = 204.0794) in MS/MS ion fragmentation spectrum of the second run. Glycopeptide candidates were further filtered manually by matching fragment ions with predicted glycopeptide sequences. The retention time of a positive candidate was used to determine the retention time for all other glycopeptides in the results of the first run. All other glycopeptides were determined by matching the observed molecular weights with predicted ones in the results of the first run. The relative abundance of each glycopeptide was calculated by its corresponding peak areas of extracted ion chromatograph.

### 4.6. Cell Lines and Cell Culture Conditions

The hereditary spherocytosis cell line Wil2-S was obtained from the American Type Culture Collection (ATCC, Manassas, VA, USA). Wil2-S cells were cultured in RPMI (Roswell Park Memorial Institute medium, Corning, NY, USA) media supplemented with 10% fetal bovine serum (VWR, Radnor, PA, USA). Cells were seeded in culture flasks at a density of 1×10^5^ cells/mL, incubated at 37 °C with 7% CO_2_, and sub-cultured when the cell density reached approximately 1.5–2×10^6^ cells/mL.

### 4.7. CD20 Binding Assay

The binding of plant-made rituximab to target cells (Wil2-S) was determined with flow cytometry analysis using a Becton Dickinson Accuri C6 flow cytometer. Wil2-S lymphoma cells at 1×10^6^ cells/mL were incubated with different concentrations of Rituxan^®^ (Genentech, South San Francisco, CA, USA), or plant-made rituximab treated either with 5 µM, 0.25 µM or no kifunensine for 45 min at 4 °C. Cells were washed and incubated with FITC conjugated anti-human IgG Fc (BioLegend, San Diego, CA, USA) in phosphate-buffered saline (PBS) with 2% fetal bovine serum (FBS). FITC-labeled Mouse IgG2a, kappa (BioLegend, San Diego, CA, USA) was used as an isotype control. Cells were analyzed by flow cytometry after briefly washing them with PBS containing 2% FBS. 7-AAD (7-amino-actinomycin D) exclusion dye was used for the quantification and segregation of dead cells in each sample. Cell Quest data acquisition software (BD Biosciences, San Jose, CA, USA) and Flowjo FACS (Fluorescence-activated cell sorting) analysis software (Tree Star Inc., Ashland, OR, USA) were used to derive data plots. Cell labelling and Flowjo FACS analysis was as described earlier [45].

### 4.8. Antibody-Dependent Cell-Mediated Cytotoxicity (ADCC) Assay

The ADCC reporter assay was performed using Wil2-S cells as targets along with Jurkat-CD16 reporter cell lines. Two reporter cell lines stably expressing the FcγRIIIa receptor, V158 (high affinity) or F158 (low affinity) variants (Promega, Madison, WI, USA) were used. Wil2-S cells were plated in a 96-well white bottom assay plate at 5000 cells per well. Serial dilutions of test antibodies were added to the plates containing the target cells and incubated at 4 °C for 15 min to allow opsonization. Jurkat-CD16 reporter cells were then added to assay plates already containing Wil2-S cells and antibodies. The final concentration of antibodies ranged from 2 to 0.0003 µg/mL following several 3-fold dilutions. The effector:target cell ratio was 10:1. After a 6 h. incubation at 37 °C, One-Glo™ Luciferase Assay Reagent (Promega, Madison, WI, USA) was added and luminescence was determined using a Gen5 microplate reader. Samples and controls were tested in triplicate, and the mean reporter signals of sample dilutions in Relative Luminescence Units (RLU) were plotted against the antibody concentration. Antibody independent cellular cytotoxicity (AICC) was measured in wells containing target and effector cells without antibodies. GraphPad prism software (GraphPad, La Jolla, CA, USA) was used to plot normalized RLU versus Log10. The half maximal effective concentration (EC_50_) values of plant-made rituximab and Fc variants were derived as dose responses obtained from non-linear regression curves. Fold of induction was calculated by taking the ratio of background subtracted induced RLU and background subtracted untreated control.

## 5. Conclusions

Glyco-engineering methods represent promising means to enhance biological activities of therapeutic glycoproteins. In this study, we evaluated the effectiveness of α-mannosidase-I inhibitor kifunensine, to modulate the metabolic pathway of the *Nicotiana benthamiana* transient expression platform. We demonstrated that 0.375 µM of kifunensine applied to the infiltration solution was sufficient to block the trimming of Man_9_ and Man_8_ oligosaccharides and resulted in the production of completely afucosylated rituximab, an anti-CD20 antibody. Oligomannose rituximab glycoforms did not possess any plant-specific α(1,3)-fucose and β(1,2)-xylose and showed an increase antibody-dependent cell-mediated cytotoxicity (ADCC) when compared to Rituxan^®^ or rituximab with plant complex-glycans. In addition, kifunensine modulated the plant N-glycosylation pathway without affecting plant growth or protein expression. Incorporating kifunensine into the *N. benthamiana* transient expression system, a cost-effective and scalable expression system, provides a simple alternative to the generation of transgenic plants, both expensive and time-consuming. This technology is applicable to many therapeutic glycoproteins for which oligomannose glycosylation offers a therapeutic advance, like the anti-cancer antibody rituximab.

## Abbreviations

mAb	Monoclonal antibody
ADCC	antibody-dependent cell-mediated cytotoxicity
Man_8_	mannose-8 glycan
Man_9_	mannose-9 glycan
EC_50_	half maximal effective concentration
PGC	polypropylene glycol
